# The Regulatory Role of Rolipram on Inflammatory Mediators and Cholinergic/Adrenergic Stimulation-Induced Signals in Isolated Primary Mouse Submandibular Gland Cells

**DOI:** 10.1155/2016/3745961

**Published:** 2016-04-07

**Authors:** Dong Un Lee, Dong Min Shin, Jeong Hee Hong

**Affiliations:** ^1^Department of Physiology, College of Medicine, Gachon University, 191 Hambakmeoro, Yeonsu-gu, Incheon 406-799, Republic of Korea; ^2^Department of Oral Biology, BK21 PLUS Project, Yonsei University College of Dentistry, Seoul 120-752, Republic of Korea

## Abstract

Exposure to bacterial lipopolysaccharides (LPS) induces inflammatory signals in salivary glands. We investigated the regulatory role of phosphodiesterase 4 (PDE4) inhibitor rolipram on inflammatory mediators and cholinergic/adrenergic stimulation-induced intracellular Ca^2+^ signaling in salivary acinar and ductal cells. Submandibular gland (SMG) expressed PDE4A through 4D mRNA and PDE4 was localized in the luminal membrane of SMG. LPS induced Ca^2+^ signaling and ROS production in SMG. Treatment with rolipram blocked LPS-induced Ca^2+^ increase and ROS production. The application of histamine evoked Ca^2+^ signals and ROS production, which were attenuated by rolipram in SMG cells. Moreover, LPS-induced NLRP3 inflammasome and cleaved caspase-1 were inhibited by rolipram. The inhibitory role of rolipram in ROS-induced Ca^2+^ signaling was mainly observed in acinar cells and not in ductal cells. Rolipram also protected SMG acinar but not ductal cells from LPS-induced cell membrane damage. In the case of cholinergic/adrenergic stimulation, carbachol/isoproterenol-induced Ca^2+^ signals were upregulated by the treatment of rolipram in SMG. In the case of cAMP-dependent ductal bicarbonate secretion by rolipram, no effect was observed on the modulation of ductal chloride/bicarbonate exchange activity. Rolipram could suppress the inflammatory signals and could be a potential therapeutic strategy against LPS-induced inflammation to protect the salivary gland cells.

## 1. Introduction

The secretion of saliva is mediated by the autonomic nervous system, which modifies the protein composition of saliva and triggers fluid secretion. The neuronal release of acetylcholine from parasympathetic nerves plays a central role in inducing salivary fluid secretion from the salivary glands [1]. Salivary acinar and ductal functions are regulated by numerous molecular components and mainly involve the activation of Ca^2+^ and cyclic adenosine monophosphate (cAMP) signaling. Phosphodiesterase (PDE) is an important enzyme, responsible for the regulation of intracellular cAMP and cyclic guanosine monophosphate (cGMP) level. It is well established that enhanced cAMP concentration activates cAMP-dependent kinase and subsequently triggers exocytosis [2]. PDEs are classified into at least 11 families based on affinity, specificity, and amino acid sequences [3, 4]. In the submandibular gland (SMG), PDE isoforms PDE1–PDE5 are expressed in an age-dependent or tissue-specific manner in rodents [5]. PDE4 is broadly distributed throughout the body and identified with four gene products and multiple splice variants [5, 6]. The hydrolytic activity of PDEs is important for the modulation of various cellular functions. For example, the involvement of PDE4 has been studied in the release of amylase from parotid acinar cells [7]. Targeting of PDE5 is associated with *β*-adrenergic receptor-stimulated mucin secretion in SMG cells [8]. However, in tissues except the salivary tissue, the functional significance of PDE isoforms with differential expression and specificity for any species or any tissue is not completely understood.

Lipopolysaccharides (LPS) are characteristic components of the bacterial cell wall and stimulate host cells of the innate immune system via the Toll-like receptor 4 (TLR4), a member of the Toll-like receptor protein family. TLR signaling is associated with the adaptive immune system and the initiation of inflammatory responses. Enhanced TLR4 expression is involved in Sjögren's syndrome (SS) [9] and inflammatory intestinal bowel diseases such as Crohn's disease [10]. Moreover, we previously provided evidences that TLR4 signaling is critically involved in the proinflammatory cytokine expression in gingival fibroblasts and periodontal ligament fibroblasts [11, 12]. Recently, Dusad et al. demonstrated that repetitive intranasal inhalant exposures to LPS resulted in significant bone deterioration [13]. Microbial infection and noninfectious stimuli trigger the activation of inflammasome, the protein complex consisting of NOD-like receptors (NLRs) family, pyrin domain containing-3 (NLRP3, known as cryopyrin, CIAS1, or NALP3), apoptosis-associated speck-like protein containing a CARD (ASC), and caspase-1, as the critical components of innate immune response [14]. The activated inflammasome complex leads to the secretion of proinflammatory mediators such as interleukin-1*β* (IL-1*β*) and IL-18, which cause cell damage [15]. It has also been reported that NLRP3 inflammasome-P2X7 receptor complex is involved in the inflammatory response in primary SS patients [16]. However, little is known about NLRP3 inflammasome activation by pathological stimulation in salivary glands.

Oxidative stress induced by reactive oxygen species (ROS) is a major risk factor that participates in various cellular functions including salivary gland dysfunction [17]. Several studies have demonstrated that ROS has significant capacity to mediate cell apoptosis [18, 19]. The TLR4 signaling triggered by LPS enhances ROS production through NADPH oxidase [20, 21]. The stimulation of TLR4 by LPS as an oral pathogen triggers an increase in intracellular Ca^2+^ concentration ([Ca^2+^]_i_) and results in inflammatory reaction and enhanced inflammatory cytokine expression as well as NLRP inflammasome activation as mentioned above. PDE4 inhibitors have anti-inflammatory and antioxidant effect; for example, the PDE4 inhibitor roflumilast attenuates LPS-induced inflammatory mediators in macrophages [22]. Another type of PDE4 inhibitor rolipram possesses antioxidant potency against the modulation of formyl-methionine-leucine-phenylalanine- (fMLP-) induced superoxide anion release in bronchoalveolar lavage cells [23].

The functional role of PDE4 inhibitor is associated with the cAMP-dependent mechanism. The costimulation with *β*-adrenergic agonist isoproterenol and rolipram enhanced amylase secretion in parotid acinar cells [7]. Ductal fluid and HCO_3_^−^ secretion are regulated by the luminal and basolateral membrane-associated ion transporters [2]. In addition to the antioxidative effect of rolipram, the enhanced cAMP level induced by rolipram may be involved in the synergistic stimulation of neuronal input signal and ductal bicarbonate secretion. However, little is known about the expression pattern of PDE4 and the inhibitory effect of the PDE4 inhibitor rolipram on the inflammatory mediator-induced signals in salivary glands. In addition, the regulatory role of rolipram on neuronal agonist-induced signals and cAMP-mediated ductal bicarbonate secretion in salivary glands should be clarified. In this study, we investigated the multifunction of rolipram in inflammatory mediators/neuronal agonist-induced signaling and modulation of NLRP3 inflammasome. Moreover, we explored whether rolipram modulates cAMP-dependent chloride/bicarbonate exchange activity in ductal bicarbonate secretion in isolated mouse SMG cells.

## 2. Material and Methods

### 2.1. Reagents

Fura-2-acetoxymethyl ester (Fura-2-AM) and 2′,7′-bis-(carboxyethyl)-5-(and-6)-carboxyfluorescein- (BCECF-) AM were purchased from Teflabs (Austin, TX). 3-Aminobenzamide (3-AB), histamine, LPS from* Pseudomonas aeruginosa* serotype 10, rolipram, carbamyl choline chloride (carbachol), isoproterenol, hydrogen peroxide, trypsin inhibitor, sodium pyruvate, bovine serum albumin (BSA), *β*-actin antibody, and all other chemicals not mentioned here were purchased from Sigma. Phosphate-buffered saline (PBS), Pluronic F-127 (20% in DMSO), goat serum, E-cadherin antibody, ZO-1 antibody, PARP-1 antibody, and MQAE dye were purchased from Invitrogen (Carlsbad, CA). PDE4 and caspase-1 antibodies were purchased from Fabgennix (Frisco, TX) and Abcam (Cambridge, MA), respectively. Collagenase P was purchased from Roche (Basel, Switzerland).

### 2.2. Isolation of Mouse SMG Cells

All procedures for maintaining the mice and for the isolation of acini and ducts followed Gachon University guidelines and were approved by the Animal Care and Use Committee of Gachon University. SMG isolated from 30–35 g Balb C wild-type mice were washed and resuspended in physiological salt solution (PSS) containing 140 mM NaCl, 10 mM glucose, 1 mM MgCl_2_, 5 mM KCl, 10 mM HEPES and 1 mM CaCl_2_, pH 7.4, 0.02% soybean-trypsin inhibitor, 0.1% sodium pyruvate, and 0.1% BSA and kept on ice until use. Briefly, the minced SMG was incubated in physiological salt solution A (PSA) containing 2.5 mg/10 mL collagenase P (Roche) for 8 min at 37°C. The digest was washed with PSA for three times, resuspended in PSA, and kept on ice until use.

### 2.3. Measurement of Intracellular Ca^2+^ Concentration ([Ca^2+^]_i_)

Isolated SMG cells were transferred onto cover glasses and incubated with 4 *μ*M Fura-2-AM in the presence of 0.05% Pluronic F-127 for 45 min for SMG cells in PSS at room temperature in the dark and then washed for 10 min with PSS. Changes in [Ca^2+^]_i_ were determined by measuring the fluorescence intensities using dual excitation wavelengths, 340 and 380 nm, and an emission wavelength of 510 nm. Results are presented as fluorescence (F) ratios (Ratio = F_340/380_). Emitted fluorescence was monitored using a CCD camera (Photometrics, AZ) attached to an inverted microscope (Olympus, Japan) and analyzed with a MetaFluor system (Molecular Devices, PA). Fluorescence images were obtained at 1 sec intervals and the background fluorescence at each excitation wavelength was subtracted from raw signals. ΔCa^2+^ responses were calculated by dividing the maximum Ca^2+^ peak of agonist in the presence of rolipram by the maximum Ca^2+^ peak of agonist stimulation.

### 2.4. Intracellular pH (pH_i_) Measurements

pH_i_ was measured with BCECF at dual excitation wavelengths of 495 nm and 440 nm. BCECF fluorescence was read at emission wavelengths above 530 nm. Isolated SMG cells attached onto coverslips were loaded in the chamber with BCECF in the presence of 0.05% Pluronic F-127 for 15 min incubation with PSS at room temperature with 6 *μ*M BCECF-AM. After stabilizing the fluorescence, the cells were perfused with PSS for at least 5 min before measuring pH_i_ at 37°C. CBE activity was measured by incubating the cells with CO_2_-saturated HCO_3_^−^-buffered media to acidify the cytosol. CBE activity was initiated by perfusing the cells with Cl^−^-free HCO_3_^−^-buffered media containing 140 mM Na^+^. The emitted fluorescence was monitored with a CCD camera (Photometrics, Tucson, AZ) attached to an inverted microscope (Olympus, Japan) and analyzed with a MetaMorph system (Molecular Devices, PA). Fluorescence images were obtained at 1 sec intervals and the background fluorescence was subtracted from the raw background signals at each wavelength. CBE activity was determined from the slopes and the derivatives of the first 30–45 sec of pH_i_ increases.

### 2.5. Confocal Imaging

Experiments were performed with sliced SMG tissues and isolated SMG acinar and ductal clusters were evaluated for the localization of E-cadherin. Isolated submandibular acini and ducts were plated on glass coverslips for 5 min at room temperature prior to fixation with chilled (−20°C) methanol. After fixation, immunostaining was performed as described previously [24] using 1 : 100 dilution of the E-cadherin and ZO-1 antibodies. Briefly, the cells were incubated with the primary antibodies overnight at 4°C and after washing unbound antibodies with 5% BSA/PBS for three times, the bound antibodies were detected with goat anti-rabbit immunoglobulin G (IgG) tagged with fluorescein isothiocyanate (FITC) (E-cadherin and ZO-1) and then washed with PBS for three times. Coverslips were mounted on glass slides with Fluoromount-GTM including DAPI (Electron Microscopy Sciences, Hatfield, PA) and analyzed using a LSM 700 Zeiss confocal microscope (Germany) with ZEN software. To determine the normalized intensity of E-cadherin in each image, average intensity of E-cadherin was divided by region of interest (ROI). Images were collected from four to five separate preparations of acinar and ductal cells and results are the averages from all experiments.

### 2.6. Reverse Transcription-Polymerase Chain Reaction (RT-PCR)

Total RNA was extracted from isolated SMG cells using TRIzol extraction system from Invitrogen according to the manufacturer's instructions. Total RNA was amplified according to the manufacturer's protocol using TOPscript*™* one-step RT-PCR kit from Enzynomics (Daejeon, South Korea). The primers used are listed in Table 1. The PCR was started with a denaturation step at 95°C for 5 min, followed by 35 cycles at 95°C for 1 min, an annealing step for 1 min, an extension step at 72°C for 1 min, and a final extension step at 72°C for 10 min. The PCR products were electrophoresed on 1% agarose gels.

### 2.7. Measurement of Reactive Oxygen Species (ROS) Production

To measure the ROS production in isolated SMG cells, Oxiselect intracellular ROS assay kit with green fluorescence (Cell Biolabs, CA) was used. SMG cells were suspended in PSA solution. The cells were attached to a 96-well plate treated with 0.005% poly-L-lysine (Sigma). Cells were incubated with 100 *μ*L DCFH-DA-containing media at 37°C for 1 hr and washed with PBS for three times. After discarding last PBS, the cells were treated with 20 *μ*g/mL LPS, 100 *μ*M histamine, and 100 *μ*M H_2_O_2_ in the presence or absence of 10 *μ*g/mL rolipram for 1 hr at room temperature. Then, the cells were washed with PBS for three times and lysed with PSA solution-containing Cell Lysis Buffer (Cell Biolabs). After incubation for 5 min, cell lysate was transferred to a new 96-well plate. The DCF fluorescence of the plate was measured with VICTO3 (PerkinElmer, MA) at 485 nm/535 nm (excitation/emission) wavelengths. To calculate DCF concentrations from plate, a standard curve was plotted as per the manufacturer's instructions (Cell Biolabs). The value of absorbance was substituted for the *y*-variable in the equation of standard curve.

### 2.8. Intracellular Cl^−^ (Cl^−^_i_) Measurements

Intracellular Cl^−^ was evaluated from N-(ethoxycarbonylmethyl)-6-methoxyquinolinium bromide (MQAE) fluorescence. The SMG ductal cells on the coverslip were loaded with MQAE by 30 min of incubation at room temperature in bath solution containing 5 mM MQAE and were washed by perfusion with NaCl-based solution until stabilization of the baseline signal. The MQAE fluorescence was recorded for at least 3 min to obtain the baseline and then switched perfusion solution with 0 mM Cl^−^ (0Cl^−^). Then 126 mM Cl^−^ (126Cl^−^) containing HCO_3_^−^ bath solution was added back. MQAE fluorescence was recorded at an excitation of 360 nm and light emitted at a wavelength higher than 530 nm was collected with a CCD camera (Photometrics). Images were analyzed with a MetaMorph system (Molecular Devices).

### 2.9. Western Blotting

SMG cells were isolated and stimulated with indicated components for 1 hr. Cell lysates were prepared in lysis buffer (containing 20 mM Tris, 150 mM NaCl, 2 mM EDTA, 1% TritonX-100, and a protease inhibitor mixture) by passing 15–18 times through a 27-gauge needle after sonication. The lysates were centrifuged at 11,000 ×g for 20 min at 4°C, and protein concentration in the supernatants was determined. Proteins were denatured by heating in SDS sample buffer at 37°C for 30 min. The 30 *μ*g heated protein samples were subjected to SDS-PAGE and transferred to methanol-soaked polyvinylidene difluoride (PVDF) membranes. Transferred proteins on PVDF membranes were visualized with caspase-1 (Abcam) and *β*-actin (Sigma) antibodies by enhanced luminescent solution (Thermo Scientific).

### 2.10. Statistical Analyses

Data from the indicated number of experiments were expressed as mean ± SEM. Statistical significance was determined by analysis of variance in each experiment. A value of ^*∗*^ *P* < 0.01 was considered statistically significant.

## 3. Results

### 3.1. Rolipram Inhibits LPS- and Histamine-Induced [Ca^2+^]_i_ Signaling in Mouse SMG Acinar Cells

**Figure 1:**
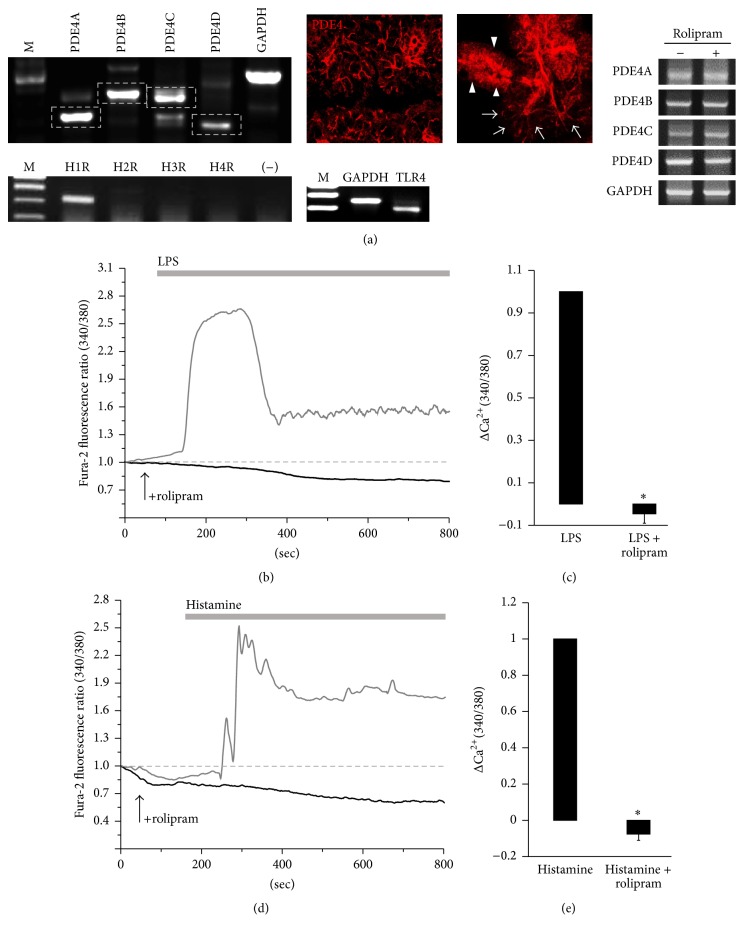
Rolipram inhibits LPS- and histamine-induced [Ca^2+^]_i_ signaling in mouse SMG acinar cells. (a) mRNA expression of PDE4 subfamily 4A through 4D and localization of PDE4 (red) in SMG tissue (left) and isolated cells (right). Arrow heads (duct) and arrows (acini). mRNA expression of TLR4 and histamine receptors (H1R to H4R) in SMG cells and expression of PDE4A through 4D in the presence of rolipram. (b) Changes in [Ca^2+^]_i_ induced by 20 *μ*g/mL LPS (gray trace) and pretreatment with 10 *μ*g/mL rolipram (black trace including arrow which indicates rolipram-stimulated time course) in SMG acinar cells. The traces are averaged trace (*n* = 4). The upper bars indicate the extracellular solutions applied to the cells. (c) Analysis of LPS-induced maximum [Ca^2+^]_i_ peak as determined using R340/380 fluorescence ratios (^*∗*^ *P* < 0.01) and means ± SEMs. (d) Changes in [Ca^2+^]_i_ induced by 100 *μ*M histamine (gray trace) and pretreatment with 10 *μ*g/mL rolipram (black trace including arrow which indicates rolipram-stimulated time course) in SMG acinar cells. The traces are averaged trace (*n* = 3). The upper bars indicate the extracellular solutions applied to the cells. (e) Analysis of histamine-induced maximum [Ca^2+^]_i_ peak as determined using R340/380 fluorescence ratios (^*∗*^ *P* < 0.01) and mean ± SEMs.

To evaluate the inhibitory role of rolipram in inflammatory mediator signaling, RT-PCR was used to assess the expression of PDE4 subfamily, TLR4, and histamine receptors (HR) in mouse SMG cells. Primarily isolated SMG acinar cells expressed PDE4A through PDE4D, TLR4, and H1R mRNA (Figure 1(a)). It will be of particular interest to determine the localization of PDE4, which may regulate cAMP-dependent cellular functions. Thus, we evaluated the protein expression of PDE4 in SMG tissues and isolated cells. Interestingly, PDE4 is localized in the luminal membrane of acini and ducts. Expression of PDE4 isoforms was not modulated in the presence of rolipram (Figure 1(a)). To evaluate whether the modulatory effect of rolipram was mediated by TLR4 activation in isolated SMG acinar cells, LPS-induced [Ca^2+^]_i_ measurement was performed in the absence or presence of rolipram. Pretreatment of rolipram inhibited LPS-induced [Ca^2+^]_i_ peak (*n* = 4, Figure 1(b)). Rolipram* per se* did not increase [Ca^2+^]_i_ response (data not shown). The inhibited [Ca^2+^]_i_ response by rolipram is depicted in Figure 1(c). These results show that LPS-triggered [Ca^2+^]_i_ response significantly (*P* < 0.01) decreased in the presence of rolipram. Similarly, rolipram inhibited histamine-evoked [Ca^2+^]_i_ response (*n* = 3, Figures 1(d) and 1(e)). These results show that rolipram has strong inhibitory effect on the inflammatory mediator-induced [Ca^2+^]_i_ signals.

### 3.2. Rolipram Prevents H_2_O_2_-Induced [Ca^2+^]_i_ Signals and Intracellular ROS Production in SMG Acinar Cells

**Figure 2:**
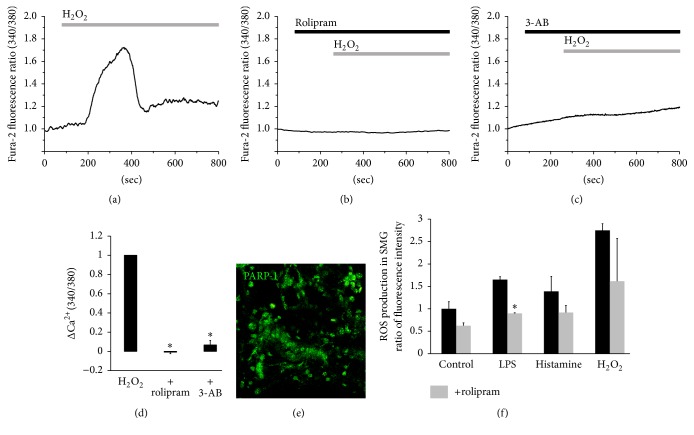
Rolipram prevents H_2_O_2_-induced [Ca^2+^]_i_ signals and intracellular ROS production in SMG acinar cells. (a) Changes in [Ca^2+^]_i_ induced by 1 mM H_2_O_2_. Changes in [Ca^2+^]_i_ induced by 1 mM H_2_O_2_, pretreatment with 10 *μ*g/mL rolipram (b) and with 10 *μ*M 3-AB (c). The traces are averaged traces (*n* = 4). The upper bars indicate the extracellular solutions applied to the cells. (d) Analysis of H_2_O_2_-induced maximum [Ca^2+^]_i_ peak as determined using R340/380 fluorescence ratios (^*∗*^ *P* < 0.01). (e) Expression of PARP-1 (green) in SMG tissue. (f) Effect of inflammatory mediators for 30 min in the absence or presence of rolipram (R) on intracellular ROS production in SMG cells. Results are expressed as ratio of control and mean ± SEMs are shown (*n* = 3, ^*∗*^ *P* < 0.01 compared with control).

Since inflammatory mediators can recruit ROS-mediated signal, H_2_O_2_-evoked [Ca^2+^]_i_ mobilization was evaluated in the presence of rolipram in the salivary glands. To examine the antioxidative role of rolipram, SMG acinar cells were stimulated with 10 mM H_2_O_2_ in the presence or absence of rolipram. The H_2_O_2_-induced [Ca^2+^]_i_ increase was significantly inhibited by rolipram (*n* = 4, Figures 2(a) and 2(b)). Preincubation of human salivary gland (HSG) cells with the ploy NAD^+^-metabolite ADP-ribose polymerase-1 (PARP-1) inhibitor 3-AB significantly decreased H_2_O_2_-induced [Ca^2+^]_i_ increase [25]. Similarly, in the present study, the H_2_O_2_-induced [Ca^2+^]_i_ signaling in mouse SMG cells was attenuated by 3-AB (*n* = 4, Figures 2(c) and 2(d)). PARP-1 was localized in the cytosol and predominantly in SMG cell nuclei (Figure 2(e)). These results suggest that rolipram attenuates H_2_O_2_-induced [Ca^2+^]_i_ increase in SMG acinar cells. To determine whether rolipram inhibits ROS production, whole SMG cells including ductal cells were stimulated with inflammatory mediators LPS, histamine, and H_2_O_2_ for 30 min and the DCF fluorescence intensities were measured. Rolipram inhibited LPS- and histamine-induced ROS production as well as the resting ROS level; however, rolipram moderately inhibited H_2_O_2_ stimulation (*n* = 3, Figure 2(f)).

### 3.3. Differential Role of Rolipram in SMG Acinar and Ductal Cells

**Figure 3:**
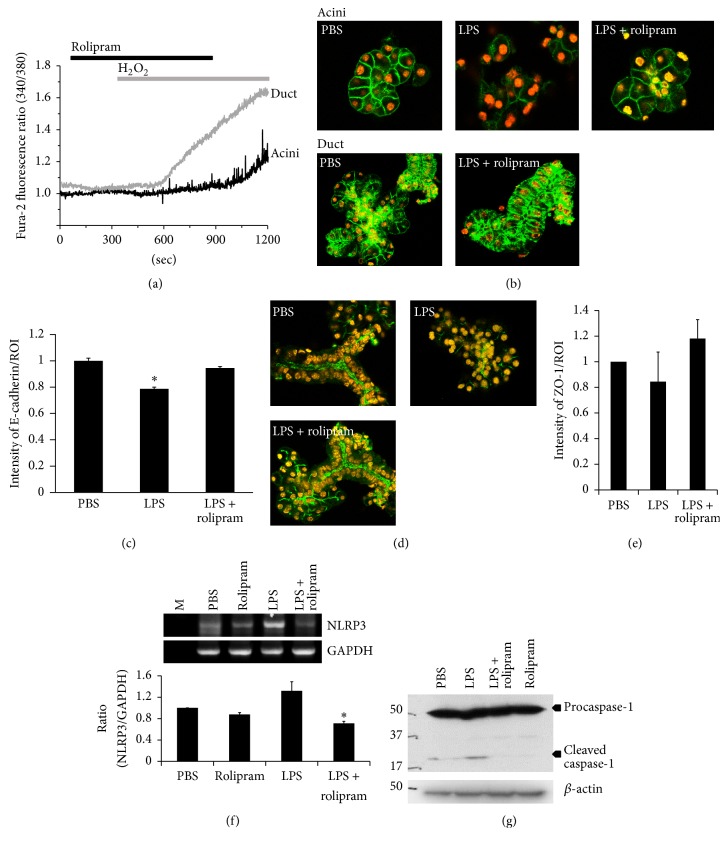
Differential role of rolipram in SMG acinar and ductal cells. (a) Changes in [Ca^2+^]_i_ induced by 10 *μ*M H_2_O_2_ and pretreatment with 10 *μ*g/mL rolipram in acinar (black trace) and ductal cells (gray trace). (b) Immunofluorescence staining patterns of E-cadherin protein (green) and DAPI staining (orange) of SMG acinar and ductal cells. The isolated SMG cells were treated with LPS in the absence or presence of rolipram for 1 hr. (c) Relative intensity of E-cadherin in plasma membrane (PM) fraction divided by the region of interest (ROI). The bars show the mean ± SEM (*n* = 3). (d) Immunofluorescence staining patterns of ZO-1 protein (green) and DAPI staining (orange) of SMG acinar and ductal clusters indicated experimental condition. (e) Relative intensity of ZO-1 divided by ROI. The bars show the mean ± SEM (*n* = 3). (f) mRNA expression of NLRP3 after the indicated stimulation for 30 min. Ratios are expressed as ratio of control and means ± SEMs are shown (*n* = 3, ^*∗*^ *P* < 0.01) compared with PBS. (g) Western blot analysis with caspase-1 after stimulation of indicated condition for 1 hr.

While rolipram significantly inhibited the inflammatory mediator-induced [Ca^2+^]_i_ response, its effect on H_2_O_2_ stimulation was not marked. Accordingly, we compared the effect of rolipram on H_2_O_2_-induced [Ca^2+^]_i_ response between acini and ductal cells. Interestingly, rolipram inhibited H_2_O_2_-induced [Ca^2+^]_i_ response of acinar cells but showed no effect on that of ductal cells (Figure 3(a)). To further demonstrate the role of rolipram as shown in Figure 3(b), the cells were stimulated with LPS to induce cell membrane damage and stained with the plasma membrane marker E-cadherin. The notable finding was that the SMG acini cell membrane was protected by rolipram in the presence of LPS (Figures 3(b) and 3(c)). It was hard to find ductal cells after stimulation with LPS (data not shown); however, in the presence of rolipram, E-cadherin and tight junction marker ZO-1 staining could be observed at the plasma and apical membrane, respectively (Figures 3(b)~3(e)). TLR4 activation by LPS and subsequent [Ca^2+^]_i_ increases are necessary for the activation of the NLRP3 inflammasome [26]. Thus, to determine the expression of NLRP3 induced by LPS treatment and the regulatory role of rolipram in expressing the NLRP3 inflammasome, SMG cells were treated with LPS for 30 min in the absence and presence of rolipram. We observed for the first time that isolated SMG cells significantly expressed NLRP3 in the presence of LPS. As shown in Figure 3(f), LPS enhanced NLRP3 mRNA expression, but rolipram attenuated NLRP3 expression. Inflammasome complex consists of NLRP3, ASC, and caspase-1 [27]. We confirmed that well-established inflammasome component caspase-1 was inhibited by rolipram (Figure 3(g)).

### 3.4. Effect of Rolipram on Synergistic Regulation of Cholinergic and *β*-Adrenergic Stimulation in SMG Cells

**Figure 4:**
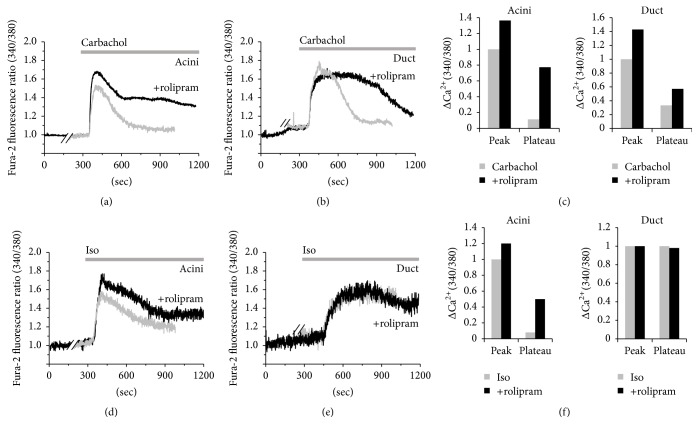
Effect of rolipram on synergistic regulation of cholinergic and *β*-adrenergic stimulation in SMG cells. Changes in [Ca^2+^]_i_ induced by 10 *μ*M carbachol (gray trace) and pretreatment with 10 *μ*g/mL rolipram (black trace) in SMG acinar (a) and ductal (b) cells. (c) Analysis of carbachol-induced maximum [Ca^2+^]_i_ peak and plateau from baseline as determined using R340/380 fluorescence ratios in acini and ductal cells. Changes in [Ca^2+^]_i_ induced by 100 *μ*M isoproterenol (gray trace) and pretreatment with 10 *μ*g/mL rolipram (black trace) in SMG acinar (d) and ductal (e) cells. All traces were averaged. (f) Analysis of isoproterenol-induced maximum [Ca^2+^]_i_ peak and plateau from baseline as determined using R340/380 fluorescence ratios in acini and ductal cells. The upper bars indicate the extracellular solutions applied to the cells.

In addition to the anti-inflammatory effect of rolipram, the role of rolipram in neurotransmitter inputs such as cholinergic/adrenergic stimulation-induced [Ca^2+^]_i_ response was evaluated in SMG acinar and ductal cells; the cells were stimulated with the muscarinic receptor agonist, carbachol, and the *β*-adrenergic agonist, isoproterenol (Iso) in the absence and presence of rolipram. In Figures 4(a)–4(d), acinar and ductal cells showed carbachol/isoproterenol-induced [Ca^2+^]_i_ peak and plateau responses except ductal isoproterenol-induced [Ca^2+^]_i_ response (Figure 4(e)). These results indicated that cholinergic/adrenergic stimulation-induced [Ca^2+^]_i_ responses were enhanced in the presence of rolipram.

### 3.5. Effect of Rolipram on Chloride/Bicarbonate Exchanger Activity

**Figure 5:**
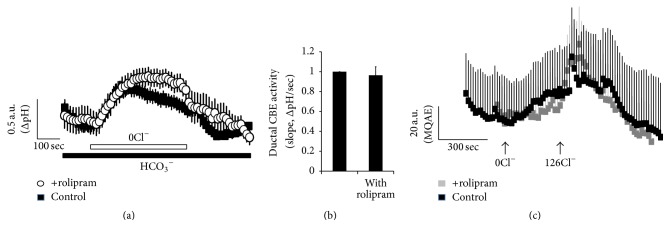
Effect of rolipram on chloride/bicarbonate exchanger activity. (a) Changes in intracellular pH (pH_i_) in the ductal cells in the presence of rolipram (open circle) and control (closed black square). (b) Ductal chloride/bicarbonate exchanger (CBE) activity. The slope of pH_i_ assessed CBE activity in the absence of Cl^−^ at the beginning of time course (30~45 sec) and height to reach maximum pH_i_ point from minimum point. The bars show the mean ± SEM (*n* = 3). (c) Changes of intracellular Cl^−^ concentration. Ductal cells were loaded with MQAE dye and fluorescence was monitored in the presence of rolipram (closed gray square) and control (closed black square). The traces were measured with arbitrary MQAE intensity every 3 sec.

The cAMP signaling pathway stimulates epithelial chloride/bicarbonate exchanger such as luminal solute carrier 26 family member 6 (SLC26A6) in the duct. Enhanced cAMP and [Ca^2+^]_i_ concentration resulted in the synergistic activation of ion transporters, which induce changes in intracellular pH (pH_i_) and directly reflect epithelial fluid secretion [25, 28]. To determine effects of rolipram on the modulation of cAMP-associated ion transporters such as anion exchanger and solute carrier transporter, we measured the chloride/bicarbonate exchanger activity in ductal cells. The chloride/bicarbonate exchanger activity was evaluated by measuring the changes in pH_i_ induced by acute chloride removal and subsequent addition of chloride (Figures 5(a) and 5(b)). Removal of chloride in the perfused solution induced intracellular alkalinization in ductal cells. The slope of the change in pH_i_ effect was measured using chloride-free bicarbonate-buffered solution. We confirmed chloride movement with chloride-sensitive dye MQAE in ductal cells (Figure 5(c)). As shown in Figure 5, the slope of pH_i_ as chloride/bicarbonate exchanger activity and chloride movement in the presence of rolipram did not show any statistical difference.

## 4. Discussion

In this study, we demonstrated the regulatory effect of PDE4 inhibitor rolipram on Ca^2+^ signaling and ROS production induced by the stimulation of inflammatory mediators in acinar and ductal cells isolated from mouse submandibular salivary glands. Additionally, various PDE4 subfamilies have been reported to be present in mouse submandibular glands and PDE4 was localized in the luminal membrane of acini and ducts. Rolipram has been developed and studied as a potent antidepressant, which elevates rat brain cAMP* in vivo* [29, 30]. PDE family members degrade cAMP, and they are expressed fundamentally in all immune cells including neutrophils, eosinophils, lymphocytes, and macrophages. The anti-inflammatory role of PDE inhibitor rolipram on immune cells is well established, whereas the role of rolipram on salivary glands has been addressed in cAMP-dependent amylase secretion [5, 7]. Regulation of inflammatory signal and cAMP-dependent electrolyte secretion by rolipram in salivary gland remains unknown. Moreover, there are no previous reports indicating the effect of rolipram on inflammatory mediators or cholinergic/adrenergic-induced signaling and cAMP-dependent bicarbonate secretion in the salivary glands.

In the present study, we focused on the inhibitory effect of rolipram on inflammatory mediator-induced [Ca^2+^]_i_ signaling and ROS production. In the presence of rolipram, LPS or histamine-induced [Ca^2+^]_i_ signaling was dramatically attenuated in mouse SMG acinar cells. In addition, H_2_O_2_-induced [Ca^2+^]_i_ signaling was inhibited by rolipram. Pretreatment of rolipram increased cAMP concentration and subsequently may activate protein kinase A (PKA), leading to reduction of ROS production and, moreover, may block the [Ca^2+^]_i_ signaling cascade. However, how to acutely attenuate [Ca^2+^]_i_ signaling by rolipram and its signaling mechanism should be clarified in coming years. LPS-induced oxidative stress was addressed by ROS through NADPH oxidase (Nox). TLR4 recruits the Nox4 and then is able to generate ROS in the form of H_2_O_2_ [31]. Sustained Ca^2+^ level triggers mitochondrial damage including increased mtROS production [32]. Although the candidate of ROS source was most likely Nox4, we cannot rule out the mitochondrial ROS source.

The TLR signaling results in the production and expression of inflammatory mediators including IL-6 and IL-8 in the salivary glands [9]. Normal human SMG cells expressed TLR1-10 mRNA and salivary glands of patients with SS showed enhanced expression of TLR2, TLR3, and TLR4 [9, 33]. Stimulation of human salivary gland cells with TLR ligands augmented the expression of inflammatory cytokines such as IL-6 and CD54 and further cell apoptosis, which means TLR signaling may be involved in pathological process in SS [9]. We provided the first evidence that TLR-mediated [Ca^2+^]_i_ signal is associated with the enhanced gene expression of NLRP3 inflammasome, which is attenuated by rolipram in SMG cells (Figure 3(f)) and cleaved caspase-1 signaling was inhibited (Figure 3(g)). LPS signaling and generation of ROS are common events in NLRP3 inflammasome activation. Although ligand of NLRP3 inflammasome is unclear, enhanced cAMP production inhibited the activation of the NLRP3 inflammasome through the direct regulatory role of cAMP on the NLRP3 complex [26]. We speculated that the increased cAMP by rolipram might inhibit the activation of inflammasome. However, further studies investigating the precise role of rolipram and the possible involvement of PDE in the inflammasome complex such as thioredoxin-interacting protein TXNIP and adaptor protein ASC should be carried out.

It is noteworthy that rolipram, which regulates intracellular cAMP levels and inhibits the inflammatory signaling, may be applied in systemic autoimmune diseases such as SS and acute bacterial infection. In addition to the regulatory role of rolipram on the inflammatory mediators in this study, rolipram enhanced the amylase release by *β*-adrenergic stimulation in parotid glands [7]. Fluid and protein secretion from the salivary gland due to signals such as neurotransmitters input are determined by coordinated spatial and temporal modulation of Ca^2+^ signaling mechanism. Moreover, the magnitude or spatial and temporal regulation of Ca^2+^ signaling is important for the stimulation and maintenance of fluid secretion [34]. Cholinergic/*β*-adrenergic stimulations by carbachol/isoproterenol-induced [Ca^2+^]_i_ signaling were enhanced in the presence of rolipram in Figure 4, suggesting that enhanced cAMP caused by rolipram and [Ca^2+^]_i_ concentration by carbachol/isoproterenol results in the synergistic activation for fluid secretion. It has been reported that amylase secretion is regulated by cAMP and [Ca^2+^]_i_, which mediate the enhanced secretion by combined stimulation [35]. Generally, high Ca^2+^ microdomain at luminal membrane is essential to drive protein and fluid secretion [24]. Localization of PDE4 at apical membrane as shown in Figure 1(a) may ensure the fidelity and rapid activation for protein secretion by regulating the local cAMP level.

Ca^2+^ and cAMP are debatably the classical second messengers that control various types of cellular homeostasis [36]. The cAMP pathway augments Ca^2+^ signals by phosphorylation of IP_3_ receptors [37]. The Ca^2+^ signal itself stimulates Ca^2+^-dependent adenylyl cyclases [36]. Synergism of Ca^2+^ and cAMP signaling induces epithelial fluid secretion [28]. Neurotransmitter signaling such as cholinergic and muscarinic receptor activation might be integrated with synergism to control the physiological response through cAMP-PKA mechanism, whereas the signaling through histamine or LPS did not mediate in this mechanism. In salivary glands, histamine stimulation transfers its signal through H1 receptor, which is independent signaling with PKA and the LPS signal was inhibited by PKA in the pretreatment with rolipram. Otherwise, it is possible that the upstream mechanism of PKA might be involved. Although the differential role of PKA in both neuronal input and inflammatory signaling pathway should be elucidated, rolipram is an effective agent, which modulates those two signaling pathways.

In the present study, we did not observe significantly altered chloride/bicarbonate exchange activity for bicarbonate secretion in ductal cells, suggesting the effect of rolipram is more associated with acinar cells than ductal cells. The cellular distribution or expression level of PDE family including PDE4 between SMG acinar and ductal cells may be different and should be correlated with their function. Although the exact expression of other PDE isoforms might be determined in the coming years, the ductal cells were dominantly damaged by oxidative stress and showed no statistical difference in the chloride/bicarbonate exchanger activity with enhanced cAMP level by rolipram, suggesting that the duct may require other PDE family members for fluid secretion or possess more sensitivity to oxidative stress than acini. Moreover, the significance of the different PDE4 isoforms in the effect of rolipram needs to be clarified. Rolipram possessed different inhibitory action on ductal versus acinar cells in the treatment with H_2_O_2_. The antioxidative capacity between ductal and acinar cells not differential inhibitory action of rolipram should be different. The differential identification of ROS scavenging system and oxidative-dependent channels should be clarified between ductal and acinar cells.

Additionally, we cannot rule out longer treatment of rolipram. It has been proposed that long-term administration up to 2 months of calcineurin inhibitors decreases antioxidant enzyme activity and alters saliva composition [38]. Similarly, the administration of high dose of PDE inhibitor ICI 153,110 for up to 6 months influenced the epithelial cell proliferation including salivary glands [39]. Accordingly, long-term administration of rolipram may result in decreased redox control, as reflected by the enhanced antioxidative role of rolipram. In the current study, although we did not examine the long-term administration of rolipram, the involvement of PDE4 was found to modulate acute TLR-mediated [Ca^2+^]_i_ signaling and ROS production.

## Figures and Tables

**Table 1:** 

Genes	Tm (°C)	Sequences (5′ → 3′)
TLR-4	50	ATG ATG CCT CCC TGG CTC
		CCG CGG TTC TCC TCA GGT C

PDE4A	58	TTC AAG CTG CTG CAA GAA GA
		TTC CTG AGG ACC TGG ATA CG

PDE4B	58	GAA CAA ATG GGG CCT TAA CA
		TTG TCC AGG AGG AGA ACA CC

PDE4C	58	CAT GCT CAA CCG TGA GTT GT
		TGG AAC GTC TTG AGG AGG TC

PDE4D	58	GGA GCT TGT CAC CTT CTT GG
		GTG GGC TTT AAG TTG CTC CA

H1R	62	GAC TGT GTA GCC GTC AAC CGG A
		TGA AGG CTG CCA TGA TAA AAC C

H2R	60	TCG TGT CCT TGG CTA TCA C
		CTT TGC TGG TCT CGT TCC T

H3R	62	TCA GCT ACG ACC GCT TCC TGT CGG TCA C
		TTG AGT GAG CGC GGC CTC TCA GTG CCC C

H4R	60	GAA TTG TCT GGC TGG ATT AAT TTG CTA ATT TG
		AAG AAT GAT GTG ATG GCA AGG ATG TAC C

NLRP-3	65	CTC TGT GAG GGG CTT CTG CAC
		GGC ACC TGG TGG TCC TGC TTC

GAPDH	58	TTA GCC CCC CTG GCC AAG
		CTT ACT CCT TGG AGG CCA TG
